# The Role of Omega- 3 Polyunsaturated Fatty Acids in Diabetes Mellitus Management: A Narrative Review

**DOI:** 10.1007/s13668-024-00561-9

**Published:** 2024-07-20

**Authors:** Sümeyra Şahin Bayram, Gül Kızıltan

**Affiliations:** 1 Faculty of Health Sciences, Nutrition and Dietetics Department, Selcuk University, Konya, Turkey; 2 Faculty of Health Sciences, Nutrition and Dietetics Department, Baskent University, Ankara, Turkey

**Keywords:** Antidiabetic, Antiinflammatory, Antilipidemic, Antioxidant, Diabetes mellitus, Omega-3 fatty acids

## Abstract

**Purpose of Review:**

Diabetes mellitus (DM) is a group of metabolic illnesses characterized by elevated levels of glucose in the bloodstream as a result of abnormalities in the generation or function of insulin. Medical Nutrition Therapy (MNT) is an essential component of diabetes management. Dietary fats are essential in both the prevention and progression of chronic diseases. Omega-3 polyunsaturated fatty acids are recognized for their advantageous impact on health. They assist in controlling blood sugar levels and lipid profile in patients with all types of diabetes. Furthermore, they reduce the occurrence of cardiovascular events and death linked to DM.

**Recent Findings:**

After evaluating the antioxidant, anti-inflammatory, antilipidemic, and antidiabetic mechanisms of omega-3 fatty acid supplements, as well as the results from randomized controlled studies, it is clear that these supplements have positive effects in both preventing and treating diabetes, as well as preventing and treating complications related to diabetes, specifically cardiovascular diseases.

**Summary:**

However, current evidence does not support the use of omega-3 supplementation in people with diabetes for the purpose of preventing or treating cardiovascular events. People with all types of diabetes are suggested to include fatty fish and foods high in omega-3 fatty acids in their diet twice a week, as is prescribed for the general population.

## Introduction

Diabetes mellitus (DM) is a collection of metabolic disorders marked by high levels of glucose in the blood due to malfunctions in the production or effectiveness of insulin. Diabetes-induced chronic hyperglycemia can lead to persistent harm, impairment, and malfunction of various organs, particularly the eyes, kidneys, nerves, heart, and blood vessels [1]. The global diabetes population was anticipated to be 537 million in 2021, with projections indicating an increase to 643 million by 2030 and 783 million by 2045 [2].

Medical Nutrition Therapy (MNT) is a vital aspect of diabetes care. Emphasizing the equilibrium of macronutrients, minimizing carbohydrate intake, reducing the glycemic index, and adopting an overall healthy eating regimen are key strategies for medical nutrition therapy in diabetes [3]. Existing evidence indicates that there is no universally recommended distribution of energy from carbohydrates, protein, and fat for individuals with diabetes. It is suggested that the allocation of macronutrients should be tailored to the individual's specific eating habits, preferences, metabolic objectives, and physical activity level [4]. The National Academy of Medicine (NAM) recommends the Acceptable Macronutrient Distribution Range (AMDR) values as follows: carbohydrates should make up 45–60% of the diet, proteins should make up 10–20%, and fats should make up 20–35% [4, 5]. Although it is acknowledged that the quality of dietary fat has a greater impact on insulin sensitivity and the risk of developing type 2 diabetes mellitus (T2DM) compared to the quantity of fat consumed [4], the American Heart Association (AHA) advises that saturated fatty acids should not contribute more than 6% of total energy intake [6].

A comprehensive review and meta-analysis of randomized controlled trials investigated the impact of dietary fat on glycemic control. The study revealed that substituting saturated fatty acids (SFAs) in the diet with polyunsaturated fatty acids (PUFAs) led to enhanced glycemic control and reduced insulin resistance [7]. Dietary fats play a crucial role in both the prevention and development of chronic illnesses. The dietary intake of omega-3 (n-3) and omega-6 (n-6) fatty acids is considered crucial in the prevention and development of chronic disorders. Omega-3 polyunsaturated fatty acids are known to have beneficial effects on health. They help regulate blood sugar levels and lipid profile in individuals with all type of diabetes by exerting anti-inflammatory, antioxidant, and antilipidemic actions. Additionally, they lower the incidence of cardiovascular events and mortality associated with diabetes [8]. In this review, we aimed to report on effects of omega-3 polyunsaturated fatty acids on diabetes and its complications’ management. This is significant because diabetes; one of the most dangerous and frequent chronic diseases of our time, is a major public health concern that endangers lives, decreases quality of life, causes critical complications, and places a significant strain on healthcare systems. The goal of diabetes prevention and treatment is to reach and maintain specific targets in blood glucose levels, blood pressure, lipid profile, and body weight through the development of health-promoting food habits. Omega-3 polyunsaturated fatty acids, which are anti-inflammatory and antioxidant, are indispensable components of nutritional models with high evidence that they have many protective and health-promoting effects against chronic diseases. Previous studies have mostly examined the relationship between omega-3 and type 2 diabetes. In this study, we examined studies with samples consisting of individuals with all types of diabetes including type 1 diabetes mellitus (T1DM), T2DM, gestastional diabetes mellitus (GDM) and prediabetes (Impaired Fasting Glucose: IFG; Impaired Glucose Tolerance: IGT).

## Methods

This study was conducted out by searching the databases "PubMed, Web of Science, ScienceDirect, Google Scholar, and Scopus" through Selcuk University Library (using the following keywords alone or in combinations: "omega-3 fatty acids," "diabetes mellitus," "antiinflammatory," "antioxidant," "antilipidemic," and "antidiabetic"). This analysis comprised randomized controlled trials published over the last decade that investigated the impact of omega-3 polyunsaturated fatty acids on diabetes prevention, management, and complications. Thus, the search was narrowed to randomized controlled trials between 2015 and 2024. After adding the inclusion criteria, the total text was 311. Exactly 103 trials were included after reading the titles. Then, a total of 75 trials were included after reading the abstract. Eventually, after reading the full texts 35 articles were included based on the quality of the studies. The inclusion criteria for the trials considered factors such as literature review, trial design, study sample, intervention period, data collection, outcomes, and limitations. Special attention was focused on recent studies.

## The Roles and Functions of Polyunsaturated Fatty Acids

Polyunsaturated fatty acids (PUFAs), which are primarily classified as omega-3 (n-3) and omega-6 (n-6) fatty acids, are essential fatty acids that are crucial for maintaining body homeostasis and cannot be synthesized internally. Through dietary consumption, these substances become part of the membranes of platelets, erythrocytes, neutrophils, monocytes, and neuronal cells [9].

Alpha-linolenic acid (ALA; 18:3 n-3) and linoleic acid (LA; 18:2 n-6) are polyunsaturated fatty acids that are considered important for the human body. This is because humans do not possess the enzymes required to add double bonds to fatty acids beyond carbons 9 and 10, which are numbered from the carboxylic acid end [10]. Arachidonic acid (ARA; 20:4 n-6), a derivative of LA and necessary in cases of LA insufficiency, together with eicosapentaenoic acid (EPA; 20:5 n-3) and docosahexaenoic acid (DHA; 22:6 n-3), generated from ALA, serve as the precursors for crucial mediators involved in the inflammatory response and several cellular functions [11]. While the human body has the ability to convert ALA to EPA and subsequently to DHA, the conversion process is inefficient. As a result, EPA and DHA can be regarded conditionally necessary. PUFAs can be found abundantly in various vegetable oils, nuts, oilseeds, and some varieties of fish [9]. ALA and LA are prevalent in plant oils, whereas EPA and DHA can be acquired from fish. ARA, a significant n-6 fatty acid, is often derived from animal sources in the diet and acts as a precursor in the production of prostaglandins (PG), thromboxanes (TX), and leukotrienes (LT) [10].

PUFAs, besides serving as energy sources, fulfill several roles inside the human body. Long-chain (LC) PUFAs play a crucial role, particularly in specialized cells and organs such the brain, retina, heart, and liver. Phospholipids are crucial elements of cell membranes, serving as essential structural components. They play a vital role in regulating the passage of substances into and out of cells, maintaining the flexibility of the membrane, facilitating cell communication, and controlling gene expression and cellular activities [9, 12]. Additionally, PUFAs have a direct impact on various metabolic pathways that serve as ligands for transcription factors like sterol regulatory element binding protein 1 (SREBP-1), nuclear factor kappa B (NF-κB), hepatocyte nuclear factor 4α (HNF-4α), and peroxisome proliferator-activated receptors (PPARs) [12].

**Fig. 1:**
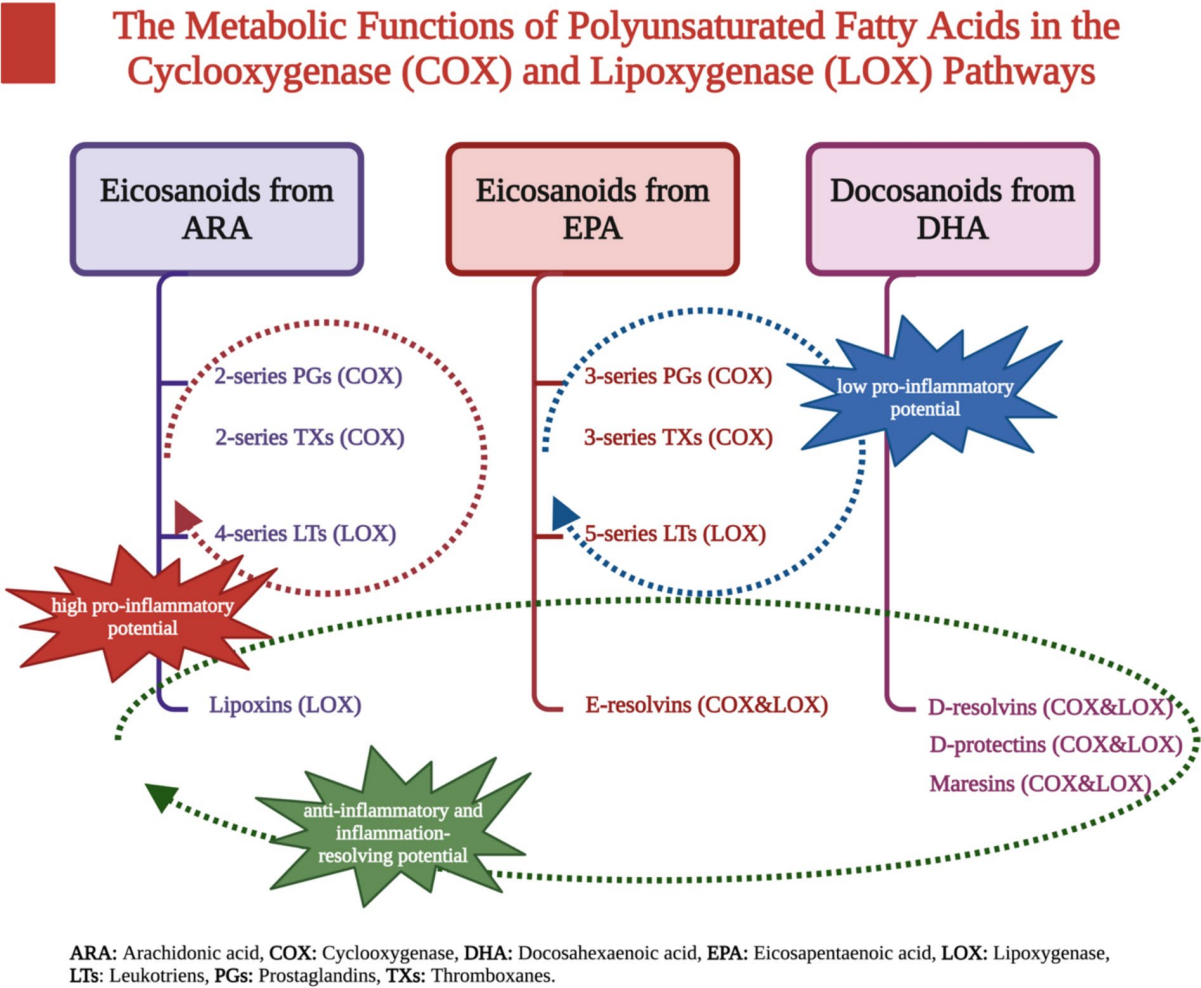
The metabolic functions of polyunsaturated fatty acids in the cyclooxygenase (COX) and lipoxygenase (LOX) pathways [14–17]

The formation of different types of pro-inflammatory and anti-inflammatory eicosanoids is mostly attributed to the actions of LA and ALA. Eicosanoids are biologically active signaling lipids that are synthesized from ARA, dihomo-gamma-linolenic acid (DGLA; 20:3 n-6), EPA, and DHA by the action of enzymes such as cyclooxygenase (COX-1 and COX-2), lipoxygenase (5-LOX and 15-LOX), and epoxygenase. In order to prevent chronic diseases and preserve good health, it is necessary to consume a balanced amount of n-6 and n-3 fatty acids. This is because these fatty acids have opposing effects on the formation of anti-inflammatory and inflammatory eicosanoids [13]. The metabolic function of PUFAs on the cyclooxygenase and lipoxygenase pathways is shown in Fig. 1 [14–17].

The recommended ratio of n-6:n-3 fatty acids for optimal health benefits is 1:1–2:1. Nevertheless, as a result of industrialization, the typical western diet may have a substantially elevated n-6:n-3 fatty acid ratio, reaching up to 20:1 [18]. PUFAs are extremely vulnerable to peroxidative assault, resulting in the deterioration of cellular membranes and the initiation of lipotoxicity mechanisms. Oxidized PUFAs, along with their resulting compounds, are associated with numerous illnesses and inflammatory responses. Research has demonstrated that oxidized lipid mediators originating from LCPUFAs, such as LA and AA, are associated with various pathological conditions, including metabolic disorders, non-alcoholic fatty liver disease (NAFLD), and cardiovascular diseases (CVD) [9].

## Omega-3 Polyunsaturated Fatty Acids

The initial double bond of omega-3 polyunsaturated fatty acids (n-3 PUFAs) is positioned at the third carbon atom counting from the methyl terminus of the fatty acid chain, so it is referred to as n-3. Omega-3 PUFAs mostly occur in an esterified state and are linked to phospholipids in the cell membrane and triacylglycerol in storage lipids. They consist of alpha-linolenic acid (ALA; 18:3 n-3), stearidonic acid (SDA; 18:4 n-3), eicosapentaenoic acid (EPA; 20:5 n-3), docosapentaenoic acid (DPA; 22:5 n-3), and docosahexaenoic acid (DHA; 22:6 n-3). SDA is the initial n-3 fatty acid produced from ALA and subsequently serves as a precursor for the synthesis of EPA, DPA, and DHA [19].

The European Food Safety Authority (EFSA) grants approval to multiple health claims related to the consumption of fish or EPA and DHA for maintaining normal levels of blood triacylglycerol, brain function, vision, heart function, and blood pressure. EFSA recommends a daily dietary intake of 250 mg EPA + DHA, 2 g ALA, and 10 g LA for the general population [20]. Governments (United Kingdom, Belgium, Netherlands, France, New Zealand and Australia) and health institutions (American Heart Association, Food and Agriculture Organization, World Health Organization, American Dietetic Association) recommend a daily consumption of 1.4–2.5 g of n-3 PUFAs, with EPA and DHA intake ranging from 140–600 mg per day, depending on the specific guidelines [21].

## The Sources of Omega-3 Polyunsaturated Fatty Acids

Plants and algae are the principal sources of ALA, which is mostly found in some seeds, nuts, and vegetable oils [19, 22]. The ALA content in fish is relatively low. For instance, in wild sardines, it is approximately 1.1% of the total muscle fats [23]. Flaxseed, chia seed, walnut, and echium seed oils are recognized as excellent sources of ALA. Flaxseed oil is rich in ALA, with a concentration of 49.2 g per 100 g. Other oils such as walnut, canola, and soybean also serve as sources of ALA [19]. Green leafy vegetables are also rich in PUFAs, specifically in the form of ALA (approximately 60–70% of the total fatty acids) [24]. Although breast milk does include ALA, EPA, and DHA, the most abundant form of n-3 PUFAs is ALA, which makes up 35% of the total [25].

SDA is present in both plants and fish. Salmon roe has a modest amount of SDA, while wild sardines have a somewhat greater amount of SDA. SDA is present in small quantities in most plants, except for Primrose and Borage plants, where it is found in quite high concentrations (about 4.73% and 4.99% of the total FA methyl esters of seeds, respectively) [23].

EPA and DHA are the primary omega-3 PUFAs found in marine sources. Conversely, DPA is typically found in fish oils at minimal concentrations. Cod, halibut, and tuna have the highest concentrations of DHA among all fish oils, accounting for 30% of the total DHA. Cod, halibut, and haddock have the highest levels of EPA, making up 15–19% of the total fatty acids. Plant rarity is highly uncommon [19, 23].

Crustaceans, cephalopods, and bivalves, in addition to fish and marine animals, are abundant sources of EPA and DHA [26]. However, fish acquire their DHA from their diet, typically from microalgae, and are unable to produce DHA from scratch. Therefore, microalgae, which serve as the main food source for wild fish, are best suited for providing DHA as an alternative. Microalgae contain omega-3 DHA with high bioavailability, along with other vital nutrients like iron, zinc, vitamin B3, vitamin B6, vitamin C, vitamin E, and magnesium. Some of these nutrients act as cofactors in the synthesis of DHA from ALA. It is asserted that it can serve as a viable and environmentally-friendly source [27].

DPA is present in significant quantities in various sources such as raw meat from ram, lamb, and sheep (13.8% and 16.7% respectively), salmon roe (5.5% of total lipids), fish oils (1.04–2.82% of European catfish oil), seal oil (2.64–4.74%), and fish muscle (1.6% of total fatty acids in wild sardine muscle) [23].

## The Roles and Functions of Omega-3 Polyunsaturated Fatty Acids

Omega-3 PUFAs have several beneficial effects on the body. They help maintain the fluidity of cell membranes, prevent inflammation, and promote the production of anti-inflammatory eicosanoids. They also reduce the secretion and activity of pro-inflammatory protein kinases, chemokines, cytokines, and growth factors. Additionally, they inhibit the production of adhesion molecules and improve the functions of vascular endothelial cells. Omega-3 PUFAs also prevent blood platelet aggregation and reduce triglyceride synthesis in the liver. When used instead of saturated fats, they can lower low-density cholesterol levels. Furthermore, they enhance glucose uptake, increase the saturation of cancer cell membranes, and promote the uptake of free radicals, which can damage cancer cells. Omega-3 PUFAs also suppress appetite by regulating the hypothalamus and affect gene expression in adipose tissue. To maintain body homeostasis, the body regulates the release of adipokines, increases fat oxidation and potentially energy expenditure through thermogenesis, activates mechanisms involved in muscle anabolism, and influences epigenetics. These processes have implications for chronic and metabolic diseases such as cardiovascular diseases, diabetes, cancer, obesity, and neurodegenerative diseases. It is claimed that it possesses multiple mechanisms that can offer protection against illnesses [13, 28, 29].

## Molecular Pathways Associated with Oxidative Stress in Diabetes Mellitus

Oxidative stress is recognized as a significant determinant in the progression of diabetes. In terms of the underlying mechanisms of disease, reactive oxygen species (ROS) and reactive nitrogen species (RNS) such as hydrogen peroxide, superoxide anion, nitric oxide, peroxynitrite, and hydroxyl radicals predominantly contribute to physiological and metabolic processes by inducing dysfunction in mitochondria. Mitochondrial failure in beta cells results in reduced capacity to produce ATP, leading to a decrease in glucose-mediated insulin secretion (GSIS), impairment of the nicotinamide adenin dinucleotide phosphate oxidase (NADPH) complex, and disruption of Ca^2+^ signaling related to neurotransmission [30].

Mitochondrial dysfunction due to oxidative stress; triggering apoptotic processes in pancreatic cells leading to the death and loss of beta cells; negative effect on metabolic pathways in beta cells and decreased insulin secretion due to damage to ATP-dependent potassium channels (K_ATP_); activation of toll-like receptors (TLRs); inhibition of nuclear transcription factors involved in insulin gene expression, such as insulin promoter factor-1 (Pdx-1) and MafA (a transcription factor); Activation of nuclear factor kappa B (NF-κb), JNK/SAPK (jun amino terminal kinase/stress-activated protein kinase), p38 mitogen-activated protein kinases (MAPK), and other prooxidative metabolic pathways are the basic molecular mechanisms that cause insulin resistance by causing dysfunction of beta cells [31, 32]. Free radicals can interfere with the normal process of insulin signal transduction, affecting several components such as insulin receptors (IR), insulin receptor substrates (IRs) 1 and 2, the phosphotidylinositol 3 kinase (PI3K) enzyme, Akt (protein kinase B), and the mammalian target of rapamycin (mTOR) pathway. As a result, oxidative stress can lead to diabetes and diabetic complications by impacting peripheral insulin sensitivity through at least five main molecular mechanisms: mitochondrial dysfunction, beta cell dysfunction, disruption of normal insulin signaling pathways, increased inflammatory response, downregulation, and/or localization of glucose transporter-4 (GLUT-4) [32].

Prolonged exposure to elevated levels of glucose and lipids activates multiple oxidative pathways, leading to impaired insulin secretion from beta cells in the pancreatic islets, insulin resistance in peripheral tissues, reduced glucose utilization in peripheral tissues, and abnormal production of glucose in the liver [33]. Multiple molecular cascades occurring in various metabolic pathways, including the glycolysis pathway, hexosamine pathway, protein kinase C (PKC) activation, polyol pathway, and advanced glycation end products (AGEs) generation, have been identified as pro-oxidative processes and are typically increased in individuals with DM [34].

The glycolysis pathway encompasses the cellular absorption of glucose and the subsequent enzymatic degradation of glucose into pyruvate and lactate by glucose phosphorylation. Glycolysis is regulated in various rate-limiting processes, including glucose uptake, glucose phosphorylation, and the conversion of fructose-6-phosphate (F-6-P) to fructose-1,6-diphosphate (F-1,6-P2), depending on the specific cell types involved. GLUT-4, glucokinase (GK), and 6-phosphofructo-1-kinase (6PFK1) play a crucial role in regulating glycolysis [35]. During hyperglycemic circumstances, an overabundance of superoxide anion radicals is generated, which hampers the body's antioxidant mechanisms and causes harm to nuclear DNA and other macromolecules. Upon the occurrence of DNA damage, the activation of the poly-ADP-ribose polymerase-1 enzyme (PARP-1) leads to the inhibition of the glyceraldehyde 3-phosphate dehydrogenase (GAPDH) enzyme. Consequently, the levels of glyceraldehyde 3-phosphate (GAP), fructose-6-phosphate, glucose-6-phosphate (G-6-P), and glucose increase and accumulate, leading to the activation of other prooxidative pathways such as polyol, hexosamine, AGE, and PKC [34].

The polyol pathway comprises two reactions: the enzymatic reduction of glucose to sorbitol, facilitated by aldose reductase (AR), and the enzymatic conversion of sorbitol to fructose, facilitated by sorbitol dehydrogenase. Additionally, this pathway involves the generation of NADH from NAD + . Chronic hyperglycemia activates the polyol pathway, which can have pathophysiological consequences. These include decreasing the ratio of NADPH to NADP + and reducing nitric oxide production. Additionally, it leads to the accumulation of sorbitol and an increase in osmotic load. Furthermore, it promotes protein glycation by elevating fructose concentration, which contributes to the development of NAFLD. Lastly, it increases the production of ROS by raising the ratio of NADH to NAD + , resulting in oxidative stress. Consequently, diabetes problems such as retinopathy, nephropathy, and neuropathy manifest [36].

The hexosamine pathway is characterized by the action of glucosamine-fructose amido-transferase (GFAT), an enzyme that controls the rate of metabolism of fructose-6-phosphate, a product of the glycolysis pathway. This enzyme converts fructose-6-phosphate into glucosamine-6-phosphate and then activates it to generate uridine diphosphate-N-acetylglucosamine (UDP-GlcNAc). In instances of chronic hyperglycemia, an abundance of fructose-6-phosphate is directed towards the hexosamine pathway, resulting in an increase in glucosamine-fructose amido-transferase (GFAT) activity. Excessive activity of the hexosamine pathway leads to alterations in gene expression and enhances the production of transforming growth factors (TGF-α and TGF-ß). These factors impede the growth of mesangial cells, stimulate the expansion of collagen matrix and thickening of the basement membrane. Additionally, they contribute to the oxidative process, resulting in the emergence of diabetes-related complications like nephropathy [34].

Prolonged elevation of blood glucose leads to increased levels of non-enzymatic glycation and the creation of AGEs. Advanced oxidation protein products (AOPPs) are a category of protein products that consist of dityrosine, which is the accumulation of oxidatively damaged albumin due to oxidative stress. AGEs and AOPPs are recognized as agents that cause tissue damage resulting from high blood sugar levels. They play a crucial role in the development of diabetes and its associated consequences [37].

Protein kinase C is a family of ten serine/threonine kinases that are involved in a wide range of signaling events associated with both normal and abnormal physiological circumstances [38]. The enzyme diacylglycerol (DAG) plays a crucial role in cellular signaling pathways that involve phosphatidyl serine and calcium [34]. The continuous and excessive activation of many PKC isoforms serves as a mechanism that mediates tissue damage caused by ROS in DM. The primary reason for this is the synthesis of DAG from glucose through triose phosphate, as elevated levels of ROS hinder the functioning of the glycolytic enzyme GAPDH. This leads to an increase in the intracellular levels of the precursor of DAG, triose phosphate, resulting in excessive production of DAG and accumulation of GAP [39]. Furthermore, it has been reported that heightened activation of the PKC pathway leads to the stimulation of ROS-producing enzymes, such as NADPH-oxidases and lipoxygenases, hence intensifying the oxidative conditions within the cells [34].

Excessive amounts of fatty acids and glucose are responsible for causing mitochondrial dysfunction, an elevation in free oxygen radicals, and insulin resistance. Hyperglycemia, excess free fatty acids, and insulin resistance in diabetic patients lead to increased oxidative stress. This is caused by the disruption of PKC and intracellular signal transduction, as well as the elevation of advanced glycation end products AGEs and activation of receptors involved in AGEs formation [40]. AGEs enhance the formation of ROS by suppressing the action of nitric oxide (NO) in the endothelium, leading to the overexpression of NF-κB and its target genes. Elevated levels of ROS hinder the normal functioning of insulin signaling, glucose and lipid metabolism, leading to oxidative stress and disturbances in the expression of adipokine genes. In addition, ROS can enhance the expression of receptors involved in the production of AGEs and pro-inflammatory receptors for their ligands, hence intensifying the inflammatory effects of these metabolites associated with stress [41].

Patients with DM experience notable alterations in lipid metabolism and structure, leading to the occurrence of lipid peroxidation. malondialdehyde (MDA) serves as the main indicator of lipid damage caused by free radicals and oxidative stress. Elevated levels of MDA in individuals with diabetes indicate that the consequent peroxidative damage may contribute to the progression of diabetic complications. The rise in lipid peroxidation serves as an indication of the decline in the protective mechanisms of both enzymatic and non-enzymatic antioxidants [42].

Alterations manifest in the functioning of superoxide dismutase (SOD), catalase (CAT), and active glutathione (SGH) enzymes in patients with DM. SOD facilitates the transformation of superoxide anion into hydrogen peroxide. It plays a crucial part in safeguarding cells and tissues from harm caused by ROS. Additionally, it swiftly interacts with NO, diminishing its biological activity and the generation of oxidative peroxynitrite radicals. CAT shields pancreatic beta cells from hydrogen peroxide-induced harm, and its reduced activity suggests prolonged oxidative stress. Furthermore, diminished GSH activity also leads to oxidative DNA damage [42, 43].

Mitochondria are functionally and anatomically linked to the endoplasmic reticulum (ER). In instances of prolonged over feeding, they experience stress and initiate the unfolded protein response (UPR), which subsequently triggers fundamental inflammatory pathways that hinder insulin function [44]. Patients who have diabetes or are obese experience persistent, mild inflammation characterized by elevated levels of inflammatory markers such as tumor necrosis factor alpha (TNF-α) and C-reactive protein (CRP) [45]. Chronic inflammation is a primary cause that greatly contributes to the development of insulin resistance, which in turn leads to the development of T2DM and its releated issues. Obesity is considered a significant risk factor for the development of T2DM due to its direct association with inflammation and the development of peripheral tissue resistance [46]. The transition from prediabetes to diabetes has been linked to the presence of both pro- and anti-inflammatory indicators, including adiponectin, the recently discovered extracellular AGE-binding receptor (EN-RAGE), interleukin-6 (IL-6), IL-13, CRP, IL-18, IL-1 receptor antagonist, and neopterin [47].

## Scientific Background

### The Antioxidant Impacts of Omega-3 Polyunsaturated Fatty Acids

Oxidative stress is a phenomenon caused by an imbalance between the production and accumulation of well-known ROS, including superoxide radicals (O2^•−^), hydrogen peroxide (H_2_O_2_), hydroxyl radicals (•OH), and singlet oxygen (^1^O_2_), as well as RNS such as nitric oxide (NO^•^), nitrogen dioxide (NO_2_^•^), and peroxynitrite (ONOO) in cells and tissues and the ability of the biological system to detoxify these reactive products [48, 49]. While they are typically generated as a result of oxygen metabolism, various factors such as environmental stressors (such as ultraviolet rays, ionizing radiation, pollutants, and heavy metals), elevated levels of transition metal ions like iron and copper ions, and xenobiotics (such as antiblastic drugs) significantly enhance the production of ROS/RNT and oxidative stress [49]. Oxidative stress is a contributing factor to cellular aging and is involved in various acute and chronic pathological conditions, including obesity, diabetes, CVD, acute and chronic kidney disease (AKI and CKD), neurodegenerative disorders, retinal degeneration, gall bladder illnesses, and cancer [50].

Lipids, DNA, and proteins are substances that can undergo modifications due to the excessive generation of free radicals. The assessment of lipid oxidation end products is a commonly employed biomarker for measuring oxidative stress [51]. The process by which ROS chemically react with lipids is widely referred to as “lipid peroxidation”. MDA, 4-hydroxy-2-nonenal (HNE), and F2-isoprostanes are generated from PUFAs by both chemical reactions and enzymatic interactions. These compounds are commonly recognized as biomarkers that indicate the presence of oxidative stress [52].

Free radicals have the ability to engage in several chemical reactions, including hydrogen abstraction, electron transfer (oxidation or reduction), addition, fragmentation and rearrangement, dimerization, and elimination, with amino acids, peptides, and proteins [53]. Proteins are susceptible to damage caused by ROS and RNS that are generated in both normal and oxidative stress situations. It is capable of experiencing oxidative alterations, including the oxidation of sulfur-containing residues in different amino acids, the addition of hydroxyl groups to aromatic and aliphatic groups, the addition of nitroso and glutathione groups to cysteine residues, the addition of chlorine groups to aromatic groups and primary amino groups, and the conversion of certain amino acid residues into carbonyl derivatives [51].

Protein carbonylation is a prevalent kind of oxidative modification. The primary process of protein carbonylation entails the direct influence of ROS, or the oxidation of amino acid side chains facilitated by metal catalysts, specifically targeting proline, arginine, lysine, and threonine. The indirect protein carbonylation pathway occurs when the amino acids lysine, cysteine, and histidine react with reactive carbonyl groups that are formed during the oxidation of carbohydrates [glyoxal (GO), methylglyoxal (MGO)] and lipids [HNE, MDA, or acrolein (ACR)]. The formation of AGEs and advanced lipoxidation end products (ALEs) occurs through a mechanism known as glycoxidation [54].

AGEs accumulate with the process of aging, and their presence is linked to the amount of carbohydrates consumed. Consequently, they are linked to conditions such as diabetes and obesity, atherosclerosis, Alzheimer's disease (AD), and renal failure. Oxidative modification can also occur in low density lipoprotein (LDL), resulting in the formation of oxidized LDL (oxLDL), which is linked to the development of atherosclerosis and cardiovascular disorders [51]. AOPPs have altered structures that resemble AGEs and are used as indicators of oxidative stress [54].

Biological systems possess enzymatic and non-enzymatic antioxidant mechanisms to counteract the harmful effects of free radicals. Endogenous antioxidants comprise enzymes such as SOD, CAT, glutathione peroxidase (GPx), as well as non-enzymatic substances like bilirubin and albumin. When an organism is subjected to elevated levels of free radicals, the internal antioxidant system is inadequate to provide complete protection. To address this deficiency, the body relies on external antioxidants obtained from food, dietary supplements, or pharmaceuticals [55].

The impact of omega-3 fatty acids on oxidative stress remains uncertain and subject to debate. Nevertheless, there is substantial data indicating that n-3 PUFAs have antioxidative effects rather than prooxidative effects [56]. The exact method by which omega-3 PUFAs can decrease oxidative stress remains uncertain. However, it is theorized that these effects may be attributed to immunomodulation and decreased activation of leukocytes. Activated immune cells are recognized for their ability to generate cytokines, such as TNF-α or IL-6, which in turn stimulate the generation of ROS. However, PUFAs have been found to decrease the synthesis of these pro-inflammatory cytokines. Furthermore, it has been indicated that EPA and DHA possess antioxidant properties and can effectively scavenge superoxide radicals, owing to their elevated levels of unsaturation [57].

Omega-3 PUFAs have the ability to decrease ROS produced by NADPH oxidase (NOX). NOX is a significant source of ROS production in 3T3-L1 adipocytes and is increased by hypoxia, inflammatory cytokines, and ER stress. Furthermore, EPA and DHA can serve as antioxidants by decreasing the generation of the oxidative stress biomarker F2-isoprostanes by the replacement of ARA in cell membranes and the subsequent reduction of its concentration [58].

Omega-3 fatty acid supplementation has been found to improve blood total antioxidant capacity (TAC) and GPx activity, while reducing MDA levels, according to a comprehensive review and meta-analysis of randomized controlled experiments. The study revealed that it had no substantial impact on the levels of NO, GSH, SOD, and CAT activities [59]. Through a systematic review and meta-analysis of randomized controlled studies involving patients with chronic renal failure, it has been discovered that omega-3 supplementation leads to a decrease in MDA levels and a significant increase in SOD and GPx activity [60]. Another comprehensive review and meta-analysis study, which analyzed randomized controlled studies, found that supplementation of omega-3 and vitamin E led to improvements in TAC and NO levels, while reducing MDA levels. Nevertheless, it was discovered that it did not induce any noteworthy alteration in the activities of GSH, SOD, or CAT [61].

## The Anti-inflammatory Impacts of Omega-3 Polyunsaturated Fatty Acids

The anti-inflammatory action of omega-3 fatty acids is primarily achieved through three ways. One effect of these substances is the reduction in the production of eicosanoid mediators (2-series PGs, 2-series TXs, 4-series LTs), which are highly pro-inflammatory and derived from ARA. Additionally, they increase the production of eicosanoid mediators (3-series PGs, 3-series TXs, 5-series LTs), which have lower pro-inflammatory effects compared to EPA. Furthermore, EPA and DHA promote the production of eicosanoid (E-resolvins) and docosanoid (D-resolvins, D-protectins, maresins) mediators, which have anti-inflammatory properties and aid in resolving inflammation [14, 15].

EPA and DHA have anti-inflammatory effects that involve several mechanisms. These include preventing the movement of white blood cells towards inflammation sites, reducing the expression of molecules that promote cell adhesion, disrupting the structure of lipid rafts, inhibiting the activation of a protein called NF-κB, activating transcription factors that have anti-inflammatory properties like PPAR-γ, and stimulating a G protein-coupled receptor called (GPCR120) by binding to it as a ligand [16].

The enzymatic oxidation of ARA, EPA, and DHA results in the production of lipoxins, resolvins, protectins, and maresins. These substances are classified as specialized pro-resolving lipid mediators (SPMs) [17]. Resolvins derived from EPA (E-series) and DHA (D-series), as well as protectins derived from DHA, function as anti-inflammatory substances by suppressing the movement of neutrophils across endothelial cells and the generation of chemokines, neutrophils, and cytokines such as IL-1β and TNF-α [62]. Resolvin E1 enhances the process of macrophages engulfing dying polymorphonuclear neutrophils and has a strong effect on regulating the expression of adhesion molecules (namely L-selectin) on pro-inflammatory leukocytes [63].

Maresins, a novel class of anti-inflammatory agents, exhibit robust protective properties against inflammation, oxidative stress, and illnesses associated with the immune system. Maresins primarily exert their effects on cells by enhancing the polarization and phagocytosis of M2 macrophages, suppressing the invasion of neutrophils, and stimulating the generation of Treg cells. Maresins suppress the production of proinflammatory cytokines, including IL-6, TNF-α, and IL-1β, by TLR4, MAPK, and NF-κB signaling pathways under different inflammatory conditions. Maresin 1 promotes the transformation of naive T cells into Treg cells by preventing their transformation into T-helper 1 (TH1) and T-helper 17 (TH17) cells [64].

The ANCHOR-ITT research, which involved adult patients at high risk of CVD and had controlled levels of triglycerides and LDL cholesterol by statin medication, found that the administration of icosapent ethyl resulted in a substantial 17.9% reduction in the primary endpoint of hs-CRP (high-sensitivity CRP) levels [65]. A study including HIV patients with elevated plasma triglyceride levels found that omega-3 supplementation resulted in a notable reduction in plasma hs-CRP, ARA, TNF-α, and MCP-1 (monocyte chemoattractant protein-1) levels [66]. A meta-analysis study was conducted to assess the effects of EPA and DHA on inflammation biomarkers by examining randomized controlled studies. The study indicated that the effects of EPA and DHA on plasma CRP, IL-6, and TNF-α were comparable to each other [67].

## The Antilipidemic Impacts of Omega-3 Polyunsaturated Fatty Acids

Lipoprotein lipase (LPL) plays a crucial role in lipid metabolism by acting as a limiting factor enzyme that breaks down plasma triglyceride-rich lipoproteins, such as chylomicrons and VLDL, and generates non-esterified fatty acids and 2-monoacylglycerol. Hence, the regular functioning and activity of LPL are crucial in order to uphold a harmonious metabolism of plasma triglyceride (TG) [68]. Lipoprotein lipase function: The regulation of this process involves several important proteins, including activators like insulin, apolipoprotein C-II, and apolipoprotein A-V, as well as inhibitors such apolipoprotein C-III and angiopoietin-like proteins 3 and 4 (ANGPTL 3/4) [69].

Diabetic dyslipidemia (DD) is a prominent characteristic of diabetes that is strongly and directly linked to macrovascular problems. The primary atypical lipoprotein profile observed in DD is characterized by elevated TG levels and reduced high-density lipoprotein (HDL) cholesterol levels [70]. Hypertriglyceridemia is strongly linked to the occurrence of obesity, metabolic syndrome, and diabetes. For instance, studies indicate that over half (about 50%) of individuals diagnosed with type 2 diabetes also experience hypertriglyceridemia [71].

Elevated levels of TG are caused by excessive synthesis and reduced elimination. Lipoprotein abnormalities frequently arise due to reduced inhibition of hormone-sensitive lipase, leading to an impact on the functioning of LPL, cholesterol ester transfer protein (CETP), phospholipid transfer protein (PTP), endothelial lipase (EL), hepatic lipase (HL), and the flow of fatty acids to the liver. It is shown that there is a connection with insulin resistance (IR), which results in an increase in (very low-density lipoprotein) VLDL production [70].

Research indicates that omega-3 fatty acids have the ability to lower both fasting and postprandial TG levels, with the extent of reduction depending on the dosage administered. This effect on TG is attributed to many underlying mechanisms [72]. Within these mechanisms, it serves various purposes, including the reduction of hepatic lipogenesis, the decrease in hepatic VLDL and apolipoprotein B-100 synthesis, the promotion of beta oxidation of fatty acids, the inhibition of crucial enzymes involved in hepatic TG synthesis, and the enhancement of lipoprotein lipase expression [73]. Moreover, research has shown that omega-3 fatty acids control triglyceride levels by influencing the function of different nuclear receptors, such as sterol SREBP, liver X receptor-alpha (LXRα), retinoid X receptor-alpha (RXRα), farnesoid X receptor (FXR), and PPARs [74].

SREBPs are transcription factors that control the expression of genes related to lipid synthesis and play a role in multiple physiological and pathological processes [75]. Omega-3 fatty acids decrease the production of fat in the liver by inhibiting the activity of SREBP-1c. It is believed to result in a reduction in the production of VLDL, fatty acids, and TG producing enzymes [76]. The suppressive effect of omega-3 fatty acids on SREBP-1c expression is accomplished by inhibiting the binding of the LXR/RXR heterodimer to the LXR response elements (LXREs) in the SREBP-1c promoter, which is a critical step for SREBP-1c expression [77].

PPARs are a subset of three transcription factors that can be activated by ligands and are classified under the superfamily of nuclear hormone receptors. Thus far, three distinct PPAR isoforms have been discovered in mammals: PPAR-α, PPAR-β/δ, and PPAR-γ. These isoforms regulate a set of genes that are responsible for adipogenesis, lipid metabolism, inflammation, and the maintenance of metabolic homeostasis [78]. PPAR-α enhances fatty acid oxidation in the liver, fat, heart, and skeletal muscle. On the other hand, PPAR-γ facilitates the storage of fat cells as triglycerides in adipocytes and triggers the β-oxidation of PPAR-β/δ fatty acids [74]. PUFAs metabolites and eicosanoids, including prostaglandins and leukotrienes, have the ability to function as ligands for PPARs. Activation of PPAR-α by n-3PUFAs decreases the production of new fats by inhibiting the synthesis of fatty acids and may also enhance the breakdown of fatty acids by oxidation [28]. The decline in enzymatic activity responsible for TG synthesis hampers the release of non-esterified fatty acids from adipose tissue and the availability of apoprotein B, potentially leading to a decreased release of very VLDL-TG. Furthermore, it has been reported that EPA and DHA n-3 fatty acids enhance the removal of TG by chylomicrons and VLDL particles by inhibiting the production and release of VLDL and TG in the liver [79].

A randomized trial was undertaken on adult patients with hyperlipidemia to investigate the effects of omega-3 fatty acids, delivered both through food and as a supplement. The results showed a significant reduction in total cholesterol (TC), non-HDL-C, TG levels, and the TC/HDL-C ratio. Fish consumption through food has been shown to have a greater impact on these parameters [80]. The ROMANTIC research, which focused on individuals with hypercholesterolemia and high CVD risk as per the National Cholesterol Education Program Adult Treatment Panel III (NCEP:ATP III) guidelines, found that the group getting omega-3 supplements had a significantly greater increase in TG and non-HDL-C levels compared to the group receiving rosuvastatin [81]. The STRENGTH trial, which focused on adult patients with a high risk of CVD, found that the administration of omega-3 supplements alongside regular statin therapies resulted in a notable reduction in TG and non-HDL-C levels. An observed rise in HDL-C and LDL-C levels of notable magnitude has been documented [82].

## The Antidiabetic Impacts of Omega-3 Polyunsaturated Fatty Acids

**Fig. 2:**
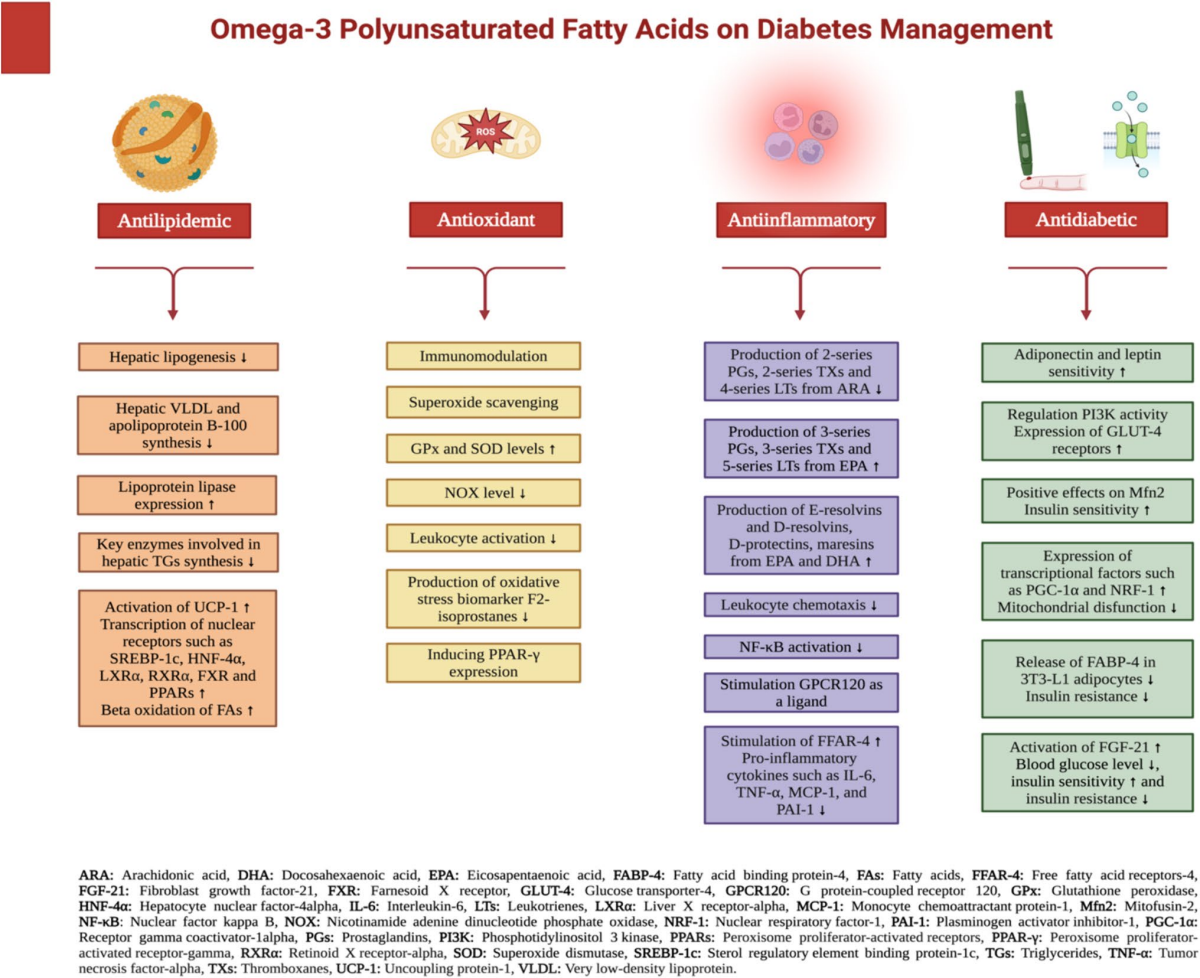
The role of omega-3 polyunsaturated fatty acids on diabetes management [13, 18, 21, 29, 44, 83–85]

The beneficial impacts of omega-3 fatty acids on prediabetes and diabetes metabolism can be attributed to their antioxidant, anti-inflammatory, antilipidemic, antiadipogenesis, insulin sensitivity-enhancing, and insulin resistance-improving properties. The functions of omega-3 fatty acids on diabetes management are shown in Fig. 2. The fundamental methods by which omega-3 polyunsaturated fatty acids (PUFAs) affect prediabetes and diabetes metabolism are outlined here [13, 18, 21, 29, 44, 83–85].

1. Omega-3 fatty acids hinder the cyclic adenosine monophosphate (cAMP) pathway, which is a secondary messenger molecule triggered by ARA. This inhibition allows protein kinase A to be activated and prevents the production of COX metabolites 2 series-PGs. These metabolites promote the formation of white adipose tissue and hinder the process of brown adipose tissue formation.
2. UCP-I (uncoupling protein-1) and PPAR-γ activation inhibits the elevation of triglycerides and the growth of adipose tissue by promoting the generation of new mitochondria, enhancing the breakdown of fatty acids, and inducing apoptosis.
3. By activating AMP-activated protein kinase (AMPK), omega-3 fatty acids reduce lipid accumulation and ROS production, therefore alleviating ER stress and increasing mitochondrial fatty acid β-oxidation. Furthermore, they exert beneficial effects on the Mitofusin-2 (Mfn2), which plays a crucial role in regulating the balance of mitochondria and the integrity of the mitochondria-associated membrane (MAM). Consequently, they enhance insulin sensitivity and safeguards its function.
4. Omega-3 fatty acids have the ability to prevent or rectify mitochondrial dysfunctions by inducing an upregulation in the expression of transcriptional factors involved in mitochondrial biogenesis, such as peroxisome proliferator-activated receptor gamma coactivator-1α (PGC-1α) and nuclear respiratory factor-1 (NRF-1).
5. Omega-3 fatty acids enhance the expression of plasma adiponectin and leptin by activating PPAR-γ, leading to improved insulin sensitivity through increased expression of GLUT-4 receptors.
6. Omega-3 fatty acids decrease the amount of fat in the muscle, maintains the normal activity of PI3K, and enhance the production and transcription of GLUT-4 receptors in the muscle, leading to improved glucose absorption by the myotubule.
7. Omega-3 fatty acids possess the ability to directly function as anti-inflammatory agents and mitigate insulin resistance by enhancing inflammation in adipose tissue. Enhancements in adipokine profiles involve an elevation in anti-inflammatory adipokines including adiponectin, an enhancement in leptin sensitivity, and a reduction in pro-inflammatory cytokines such as IL-6, TNF-α, MCP-1, and plasminogen activator inhibitor-1 (PAI-1).
8. Omega-3 fatty acids function as an agonist for various free fatty acid receptors (FFAR) that are specific to different cell types involved in both the inflammatory response and energy balance. FFAR-4 stimulation of omega-3 fatty acids inhibits the release of lipopolysaccharide (LPS)-mediated inflammatory cytokines, including as TNF-α and IL-6.
9. Fatty acid binding protein-4 (FABP-4) acts as an adipokine and is released by both macrophages and adipocytes. High levels of FABP-4 in the bloodstream are linked to obesity and insulin resistance. In 3T3-L1 adipocytes, omega-3 fatty acids have a potential to decrease the release of FABP-4.
10. Omega-3 fatty acids increase the production of fibroblast growth factor (FGF-21), which is synthesized by the liver, adipose tissue, and skeletal muscle. FGF-21 is recognized for its ability to decrease the production of glucose in the liver, lower plasma glucose levels, enhance insulin sensitivity, and promote glucose uptake in adipocytes. Consequently, the n-3 fatty acids lead to reductions in high blood sugar, elevated triglyceride levels, and plasma insulin levels, while also improving insulin resistance.
11. Omega-3 fatty acids enhance the process of removing glucose from the body and the breakdown of fatty acids, as well as the production of fatty acids, via controlling the activity of PPARs, SREBP-1c, HNF, RXR, and LXR transcription factors. Their impact on circulating triglycerides and LDL particles, membrane fluidity, and signal transduction leads to a reduction in insulin resistance.
12. Additionally, omega-3 fatty acids have beneficial effects on endothelial and vascular smooth muscle cells, inflammation and thrombus formation, plaque formation and stability, arterial stiffness, blood pressure, and cardiovascular health. They also have a good impact on atherosclerosis, which is a consequence of diabetes.

This review, which examined 35 RCTs, examined the effects of n-3 PUFAs supplementation on antidiabetic, cardiometabolic outcomes and complications of diabetes in patients with diabetes or prediabetes. Studies showing the relationship between omega-3 polyunsaturated fatty acids and antidiabetic and/or cardiometabolic outcomes are given Table 1.

**Table 1:** Studies showing the relationship between omega-3 polyunsaturated fatty acids and antidiabetic and/or cardiometabolic outcomes

**Author, year (reference)**	**Type of study**	**Type of intervention**	**Time**	**Sample size**	**Population**	**Antidiabetic effects of omega-3**
Lalia et al. 2015 [86]	Randomized controlled	**Control group:** 4.8 g oleate/d **Study group:** 4.2 g n-3 (3.9 g EPA + DHA)/d	6 months	31 (M:8 F:23)	Insulin resistant BMI: > 25 kg/m^2^ Mean age: 32.6 ± 2.5 vs. 35.3 ± 2.9	Significant decrease in TG No significant changes in FBG, insulin, HOMA-IR, TC, LDL-C, HDL-C, CRP, leptin, adiponectin, IL-6
Gharekhani et al. 2016 [87]	Randomized controlled	**Control group:** 1.8 g liquid paraffin/d **Study group:** 1.8 g flaxseed oil/d	4 months	45 (M:25 F:20)	T2DM patients on hemodialysis Mean age: 57.2 ± 15.2 vs. 56.8 ± 13.1	Significant decrease in TG**,** TC, HDL-C No significant changes in FBG, insulin, HOMA-IR, LDL-C, CRP, leptin, adiponectin
Elajami et al. 2017 [88]	Randomized controlled	**Control group:** no n-3 supplementation **Study group:** 1.86 g EPA/d and 1.5 g DHA/d	12 months	262 (M:217 F:45)	T2DM, Stable CAD, normal renal function, BMI: ≥ 27 kg/m^2^ Mean age: 63.3 ± 7.6	Significant decrease in TG for diabetics No significant changes in FBG, HbA1c, HDL-C for diabetics
Foster et al. 2017 [89]	Randomized controlled	**Control group:** corn/soy oil **Study group:** 800 mg DHA/d	During pregnancy	72 (F:72)	Pre-gravid BMI: ≥ 30 kg/m^2^ or diagnosed as GDM women at 25–29 weeks gestational age Mean age: NK	No significant changes in babies z scores of birth weight, height and body weight according to height No significant changes in BMI, body weight, height, arm circumference and arm skinfold thickness at two and four year
Hames et al. 2017 [90]	Randomized controlled	**Control group:** 4.2 g oleate/d **Study group:** 4.2 g n-3 (3.9 g EPA + DHA)/d	6 months	21 (M:6 F:15)	Insulin resistant, overweight or obese adults Mean age: 34.0 ± 9.0 vs. 36.0 ± 11.0	No beneficial effect on adipose tissue lipolysis or inflammation
Horvaticek et al. 2017 [91]	Prospective randomized controlled	**Control group:** standard diabetic diet with placebo (corn oil) **Study group:** standard diabetic diet enriched with EPA and DHA twice a day (EPA 120 mg and DHA 616 mg)	During pregnancy	90 (F:90)	Pregnant women with T1DM Mean age: NK	Significant increase in FC-peptide at third trimester in study group Significant decrease in HbA1c at third trimester in both groups
Jacobo-Cejudo et al. 2017 [92]	Randomized controlled	**Control group:** placebo **Study group:** 520 mg of n-3 PUFAs/d (320 mg EPA + 200 mg DHA)	24 weeks	54 (M:12 F:42)	T2DM, BMI: ≤ 29.9 kg/m^2^ Mean age: 48.1 ± 6.8 vs. 50.4 ± 6.3	Significant decrease in FBG, HbA1c, leptin, leptin/adiponectin ratio, TG, AI in study group Significant decrease in resistin, insulin and HOMA-IR in both groups
Jamilian et al. 2017a [93]	Randomized controlled	**Control group:** placebo **Study group:** 1000 mg/d n-3 fatty acids from flaxseed oil (400 mg ALA/d) + 400 IU/d vitamin E	6 weeks	60 (F:60)	Diagnosed as GDM women at 24–28 weeks gestational age Mean age: 30.4 ± 4.2 vs. 29.7 ± 5.5	Significant decrease in MDA and increase in TAC, NO No significant changes in GSH and hs-CRP
Jamilian et al. 2017b [94]	Randomized controlled	**(1)** 1000 mg n-3 fatty acids (360 mg EPA and 240 mg DHA) twice a day + vitamin D_3_ placebo **(2)** 50.000 IU vitamin D_3_ every 2 weeks + n-3 fatty acids placebo **(3)** 50.000 IU vitamin D_3_ every 2 weeks + 1000 mg n-3 fatty acids twice a day **(4)** vitamin D_3_ placebo + omega-3 fatty acids placebo	6 weeks	140 (F:140)	Diagnosed as GDM women at 24–28 weeks gestational age Mean age: 31.5 ± 7.0 vs. 30.7 ± 3.5 vs. 31.2 ± 4.3 vs. 30.7 ± 4.1	Significant decrease in FBG, insulin, HOMA-IR, TC, LDL-C in group 3
Mazaherioun et al. 2017 [95]	Randomized controlled	**Control group:** placebo (2700 mg paraffin/d) **Study group:** n-3 PUFAs (1800 mg EPA/d + 900 mg DHA/d)	10 weeks	85 (M:53 F:32)	T2DM BMI: 25–40 kg/m^2^ Mean age: 50.6 ± 7.2 vs. 51.1 ± 7.4	Significant decrease in MCP-1 and TG No significant changes in resistin, TC, LDL-C and HDL-C
Nasri et al. 2017 [96]	Randomized controlled	**Control group:** 1 g liquid paraffin/d **Study group:** 1.0 g flaxseed oil/d	12 weeks	60 (F:60)	PCOS women aged 18–40 y Mean age: NK	Downregulation in expression of oxidized LDLR and upregulation in expression of PPAR-γ No significant changes in expression of GLUT-1 ve Lp (a)
Poreba et al. 2017 [97]	Randomized controlled	**Control group:** placebo **Study group:** 2 g n-3 PUFAs/d (1 g EPA/d and 1 g DHA/d)	3 months	74 (M:49 F:25)	T2DM and a history of CAD Mean age: 66.7 ± 6.8 vs. 64.4 ± 6.7	No significant changes in HbA1c, insulin, peptide C, TG, TC, LDL-C, HDL-C, hs-CRP, IL-6, TNF-α, ICAM-1, VCAM-1, rvD1
Wang et al. 2017 [98]	Randomized controlled	**Control group:** four 1 g capsules corn oil/d **Study group:** four 1 g capsules fish oil/d (1.34 g EPA/d and 1.07 g DHA/d)	6 months	99 (M:35 F:64)	T2DM, waist circumference ≥ 85 cm for men and ≥ 80 cm for women Mean age: 66.3 ± 5.1 vs. 64.6 ± 5.5	Significant decrease in TG and increase in HDL-C No significant changes in FBG, insulin, HbA1c, HOMA-IR,TC, LDL-C, LDL-C/HDL-C ratio
Bowman et al. 2018 [99]	Randomized controlled	**Control group:** placebo (olive oil) **Study group:** 840 mg marine n-3 PUFAs/d (460 mg EPA + 380 mg DHA)	6 months	15,480 (M:9684 F:5796)	T2DM Mean age: 63.3 ± 9.2 vs. 63.3 ± 9.2	No significant difference in occurring serious vascular events and deaths from any cause during follow-up
Golzari et al. 2018 [100]	Randomized controlled	**Control group:** placebo (2 g paraffin/d) **Study group:** n-3 PUFAs (2 g EPA/d)	8 weeks	36 (M:9 F:9)	T2DM, BMI: < 35 kg/m^2^ Mean age: 44.7 ± 4.7 vs. 44.4 ± 3.8	Significant decrease in methionine, cystein and AI
Liu et al. 2018 [101]	Randomized controlled	**(1) Control group:** 1800 kkal diet (54% carbohydrate, 17% protein, 29% fat and n-6/n-3: 7.0–7.5, 6 g corn oil/d) **(2) LCHP group:** 1800 kkal diet (42% carbohydrate, 28% protein, 30% fat and n-6/n-3: 7.0–7.5, 6 g corn oil/d) **(3) n-3 group:** 1800 kkal diet (54% carbohydrate, 17% protein, 29% fat and n-6/n-3: 2.0–2.5, 6 g fish oil/d) **(4) LCHP + n-3 group:** 1800 kkal diet (42% carbohydrate, 28% protein, 30% fat and n-6/n-3: 2.0–2.5, 6 g fish oil/d) *6 g fish oil: 2.46 g EPA, 0.69 g DHA, 0.5 g DPA and 0.07 g ALA*	12 weeks	122 (M:61 F:61)	T2DM, HbA1c: 6.5–7.5%, SBP: 90–120 mmHg DBP: 60–90 mmHg, BMI: 18.5–23.9 kg/m^2^ Mean age: 49.7 ± 5.4 vs. 49.8 ± 5.9 vs. 50.2 ± 5.9 vs. 51.9 ± 4.8	Significant decrease in HbA1c and FBG HbA1c reduction in the LCHP + ω-3 diet group was greater than that in the LCHP and n-3 groups No significant change in HOMA-IR
Mirmasoumi et al. 2018 [102]	Randomized controlled	**Control group:** 1 g liquid paraffin/d **Study group:** 1.0 g flaxseed oil/d	12 weeks	60 (F:60)	PCOS and insulin resistant Mean age: 27.0 ± 3.2 vs. 28.4 ± 6.4	Significant decrease in insulin, HOMA-IR, TG, VLDL and hs-CRP No significant changes in FBG, NO, TC, HDL-C and LDL-C
de Boer et al. 2019 [103]	Randomized controlled	**(1)** 2000 IU vitamin D_3_/d + 1000 mg n-3 fatty acids/d (465 mg EPA/d and 375 mg DHA/d) **(2)** 2000 IU vitamin D_3_/d + placebo n-3 fatty acids/d **(3)** 1000 mg n-3 fatty acids/d (465 mg EPA/d and 375 mg DHA/d) + placebo vitamin D_3_/d **(4)** 2 placebos/d	5 years	1312 (M:703 F:609)	T2DM Mean age: 67.4 ± 7.3 vs. 67.4 ± 6.7 vs. 68.2 ± 6.7 vs. 67.5 ± 6.9	No significant changes from baseline to year 5 in eGFR and urine ACR
López-Alarcón et al. 2019 [104]	Randomized controlled	**Control group:** 1 g sunflower oil/d + hypocaloric diet **Study group:** 1.2 g EPA + DHA/d** + **hypocaloric diet	3 months	245 (M:116 F:129)	Overweight or obese adolescents Mean age: 13.6 ± 1.8 vs. 13.7 ± 2.0	No significant changes in body weight, insulin and HOMA-IR Significant decrease in TG and SBP
Raygan et al. 2019 [105]	Randomized controlled	**(1)** 2000 mg flaxseed oil/d (800 mg ALA/d) **(2)** 2000 mg fish oil/d (500 mg EPA/d and 300 mg DHA/d) **(3)** Placebo (paraffin)	12 weeks	90 (M:40 F:50)	T2DM with CDH Mean age: 64.6 ± 9.1 vs. 64.1 ± 9.3 vs. 62.0 ± 13.0	No significant changes in FBG, HOMA-IR, TG, TC, HDL-C and LDL-C Significant decrease in insulin and increase in TAC in flaxseed oil and fish oil groups Significant decrease in hs-CRP and increase in total nitrite levels in flaxseed oil
Rodríguez-Cruz et al. 2019 [106]	Randomized controlled	**Control group:** 2.9 g sunflower oil/d **Study group:** 2.9 g n-3 LCPUFA/d	6 months	28 (M:28)	Boys with DMD Mean age: 8.4 ± 2.3 vs. 7.0 ± 2.5	No significant changes in FBG and HOMA-IR
Talari et al. 2019 [107]	Randomized controlled	**Control group:** placebo **Study group:** 50 000 IU vitamin D supplements every 2 weeks + 2 × 1000 mg/d n-3 fatty acids from flaxseed oil (800 mg ALA/d)	6 months	61 (M:17 F:44)	Vitamin D-deficient T2DM with CDH Mean age: 66.4 ± 9.3 vs. 67.3 ± 8.6	Significant decrease in FBG, insulin, HOMA-IR, hs-CRP, LDL-C and increase in HDL-C
Thota et al. 2019 [108]	Randomized controlled	**Control group:** 2 × 1000 mg corn oil/d **Curcumin group:** 2 × 500 mg curcumin/d **LCn-3PUFA group:** 2 × 1000 mg fish oil/d (1.2 g EPA + DHA) **Double active group:** 2 × 500 mg curcumin/d + 2 × 1000 mg fish oil/d (1.2 g EPA + DHA)	12 weeks	64 (M:26 F:38)	Diagnosed as IFG, IGT or IFG + IGT BMI: 25–45 kg/m^2^ Mean age: 50.0 ± 2.5 vs. 55.0 ± 2.8 vs. 58.0 ± 2.5 vs. 57.0 ± 2.2	No significant changes in FBG and HbA1c in curcumin and/or LCn-3PUFA No significant changes in TC, HDL-C and LDL-C
Wang et al. 2020 [109]	Randomized controlled	**Control group:** placebo soybean powder (placebo 1) + placebo capsules (placebo 2) **Omega-3 fatty acids group:** placebo 1 + 2 g fish oil/d (1000 mg EPA + 400 mg DHA) **Plant Sterols (PS) group:** daily flour with 1.7 g plant sterols + placebo 2 **PS plus omega-3 fatty acids group:** daily flour with 1.7 g plant sterols + 2 g fish oil/d (1000 mg EPA + 400 mg DHA)	12 weeks	134 (M:65 F:69)	Diagnosed as IGR Mean age: 56.2 ± 4.1 vs. 58.9 ± 7.2 vs. 56.2 ± 7.2 vs. 55.6 ± 7.4	Significant decrease in FBG, HbA1c, HOMA-IR, TG, HDL-C in the group receiving the combined intervention Significant decrease in FBG, HOMA-IR, TG, hs-CRP in omega-3 fatty acids group
Yang et al. 2019 [110]	Randomized controlled	**Control group:** placebo **Study group:** 500 mg cod liver oil/d	4 weeks	538 (F:538)	Diagnosed as GDM women at 24–28 weeks gestational age Mean age: 27.3 ± 6.0 vs. 28.1 ± 5.4	No significant changes in FPG, 2hPG, HOMA-IR, HbA1c and lipid profiles
Golpour et al. 2020 [111]	Randomized controlled	**Control group:** 2700 mg paraffin oil/d **Study group:** 1800 mg EPA/d and 900 mg DHA/d	10 weeks	61 (M:39 F:22)	T2DM, BMI: 25–40 kg/m^2^ Mean age: 50.6 ± 7.2 vs. 51.1 ± 7.4	Significant increase in TAC Significant decrease in insulin and HOMA-IR No significant changes in FBG and HbA1C
Jamilian et al. 2020 [112]	Randomized controlled	**Control group:** sunflower oil **Study group:** 2 × 1000 mg/d *n-*3 fatty acids from flaxseed oil	6 weeks	51 (F:51)	Diagnosed as GDM women at 24–28 weeks gestational age Mean age: 28.5 ± 4.1 vs. 29.5 ± 5.0	Significant decrease in FBG, insulin, HOMA-IR, TG, TC, VLDL-C, hs-CRP and MDA Downregulation in expression of oxidized IL-1, TNF-α and upregulation in expression of PPAR-γ
Naeini et al. 2020 [113]	Randomized controlled	**Control group:** 2400 mg praffin oil/d **Study group:** 2400 mg DHA rich fish oil/d (1450 mg DHA/d and 400 mg EPA/d)	8 weeks	50 (M:23 F:27)	T2DM, BMI: 18–40 kg/m^2^ Mean age: 56.3 ± 7.8 vs. 54.7 ± 7.6	Significant increase in PPAR-γ No significant changes in mRNA expression levels of the p53 and NF-kB
O’Mahoney et al. 2020 [114]	Randomized controlled	**Control group:** 3000 mg corn oil/d **Study group:** 3300 mg/d *fish oil* (2300 mg EPA and 800 mg DHA)	6 months	20 (M:16 F:4)	T1DM, HbA1c: < 11% aged 18–65 y Mean age: 36.0 ± 17.0 vs. 32.0 ± 12.0	No significant changes in VCAM-1, ICAM-1, E-selectin, P-selectin, pentraxin-3, VEGF, TNF-α, CIMT, FMD, blood pressure, HbA1c, FPG between or within groups
Britten-Jones et al. 2021 [115]	Randomized controlled	**Control group:** 600 mg olive oil/d **Study group:** 1800 mg fish oil/d (1080 mg EPA/d and 720 mg DHA/d)	6 months	43 (M:22 F:21)	T1DM aged ≥ 18 y Mean age: 40.5 ± 19.6 vs. 48.1 ± 19.2	Significant increase in corneal nerve fiber length (CNFL) No significant changes from baseline for glycaemic control or diabetic retinopathy grading
Diaz-Rizzolo et al. 2021 [116]	Randomized controlled	**Control group:** T2DM-prevention nutritional **Study group:** T2DM-prevention nutritional + 200 g of canned sardines in olive oil per week	12 months	152 (M:84 F:68)	FBG: 100–125 mg/dl aged ≥ 65 y Mean age: 71.4 ± 5.2 vs. 71.0 ± 5.2	Significant increase in HDL-C, adiponectin and decrease in TGs, blood pressure, HOMA-IR
Liu et al. 2022 [117]	Randomized controlled	**Perilla oil (PO) group:** 1932 mg ALA/d **Fish oil (FO) group:** 858 mg EPA/d and 1032 mg DHA/d **Linseed and fish oil (LFO) group:** 630 mg EPA/d, 360 mg DHA/d and 840 mg ALA/d	6 months	150 (M:57 F:93)	T2DM with dyslipidemia Mean age: 63.8 ± 9.7 vs. 62.3 ± 7.5	Significant decrease in FBG and HbA1c in PO and LFO groups Significant decrease in TG and TG/HDL in FO group Significant decrease in insulin, C-peptid TC, Apo A1 and IL-6 in all groups
Elbarbary et al. 2023 [118]	Randomized controlled	**Control group:** placebo **Study group:** 1000 mg/d *n-*3 fatty acids	6 months	70 (M:25 F:45)	T1DM and diabetic nephropathy Mean age: 15.2 ± 2.0 vs. 14.7 ± 1.9	Significant decrease in FBG, HbA1c, triglycerides, total cholesterol, LDL-cholesterol, UACR, KIM-1 and CIMT
Kuang et al. 2023 [119]	Randomized controlled	**Control group:** 1.6 g corn oil (53.5% LA) **Fish oil (FO) group:** 1.6 g fish oil/d (29.9% EPA + 20.4% DHA) **Blue Mussel lipid extract (BMLE) group:** 1.6 g BMLE (20.7% EPA + 26.7% DHA)	2 months	133 (M:51 F:82)	T2DM, HbA1c ≥ 7% aged ≥ 40 y Mean age: 62.2 ± 9.4 vs. 59.8 ± 9.9 vs. 63.1 ± 9.4	Significant decrease in fasting insulin, HOMA-IR, TNF-a, TGs, TC, HDL-C in BMLE group
Werida et al. 2023 [120]	Randomized controlled	**Control group:** glimepirid 3 mg + placebo (corn oil and linoleic acid) **Study group:** glimepirid 3 mg + 1000 mg/d *n-*3 fatty acids (13% EPA and 9% DHA)	12 weeks	70 (M:38 F:32)	T2DM BMI: 25–35 kg/m^2^ Mean age: 50.5 ± 8.4 vs. 52.4 ± 7.6	Significant decrease in FBG, HbA1c, total cholesterol, triglycerides, LDL, HOMA-IR

**Abbreviations:** ACR: albumin to creatinine ratio; AI: atherogenic index; ALA: alpha linoleic acid; BMI: body mass index; CAD: coronary artery disease; CHD: coronary heart disease; CIMT: carotid intima-media thickness; CNFL: corneal nerve fiber length; CRP: C reactive protein; DBP: diastolic blood pressure; DHA: docosahexaenoic acid; DMD: duchenne muscular dystrophy; DPA: docosapentaenoic acid; eGFR: estimate glomerular filtration rate; EPA: eicosapentaenoic acid; F: female; FBG: fasting blood glucose; FMD: flow mediated dilation; GDM: gestasional diabetes mellitus; GLUT-1: glucose transporter-1; GSH: Glutathione; HbA1c: glycated hemoglobin A1c; HDL-C: high density lipoprotein-cholesterol; HOMA-IR: Homeostatic model assessment for insulin resistance; hs-CRP: high sensitive C reactive protein; ICAM-1: intercellular adhesion molecule; IFG: impaired fasting glucose; IGT: impaired glucose tolerance; IL-1: interleukin-1; IL-6: interleukin-6; KIM-1: kidney injury molecule; LA: linoleic acid; LCHP: low carbohydrate-high protein; LDL-C: low density lipoprotein-cholesterol; LDLR: low density lipoprotein receptor; LP(a): lipoprotein (a); M: male; MCP-1: monocyte chemoattractant protein-1; MDA: malondialdehyde; NF-kB: nuclear factor kappa B; NO: nitric oxide; PCOS: polycystic over syndrome; PPAR-γ: peroxisome proliferator-activated receptor gamma; rvD1: resolvin D1; SBP: systolic blood pressure; TAC: total antioxidant capasity; TC: total cholesterol; TGs: triglycerides; TNF-α: tumor necrosis factor alpha; T1DM: type 1 diabetes mellitus; T2DM: type 2 diabetes mellitus; UACR: urine albumin-creatinine ratio; VCAM-1: vascular cell adhesion molecule-1; VEGF: vascular endothelial growth factor; VLDL-C: very low density lipoprotein-cholesterol

The findings about glycemic control from RCTs undertaken with individuals diagnosed with T2DM are confusing. When the results are examined, no significant differences were found in HbA1c levels in four studies [88, 97, 98, 111], FBG levels in five studies [87, 88, 98, 105, 111], and HOMA-IR levels in four studies [88, 98, 101, 105]. Significant decreases were found in HOMA-IR levels in five studies [92, 107, 111, 119, 120], FBG levels in five studies [92, 101, 107, 117, 120], and HbA1c levels in four studies [92, 101, 117, 120]. The study found that the group who followed a low-carbohydrate, high-protein diet along with omega-3 experienced a greater decrease in HbA1c levels [101]. This highlights the essential importance of nutritional therapy in the management of diabetes. Upon analyzing the findings about cardiometabolic management, significant decreases were found in TG levels in eight studies [87, 88, 92, 95, 98, 117, 119, 120], and TC levels in three studies [117, 119, 120]. These differences are believed to be attributable to the size of the sample, the duration of the intervention, the mean age of the sample, and the dosage of omega-3 supplied.

When the findings from RCTs undertaken with individuals diagnosed with T1DM are examined, it was found that a six-month intervention involving the consumption of 3.3 g of fish oil by 20 T1DM patients did not lead to any significant changes in glycemic and anti-inflammatory parameters [114]. Conversely, a six-month intervention involving the administration of 1.0 g of omega-3 to 70 individuals with T1DM resulted in a significant decrease in glycemic and cardiometabolic parameters, as well as a significant improvement in complications associated with retinopathy, neuropathy, and nephropathy [118]. The reason for this could be attributed to the fact that the sample group that showed improvements in glycemic, cardiometabolic, and diabetic complications had a lower average age, resulting in a shorter duration of diabetes.

Analyzing the results of two RCTs with GDM patients reveals that a 6-week intervention involving the administration of 2.0 g of omega-3 significantly reduced insulin FBG and HOMA-IR [94, 112]. However, the study found no significant changes in FBG, HOMA-IR, and HbA1c levels after a 4-week intervention when 538 individuals with GDM consumed 0.5 g of cod liver oil [110]. This could be attributed to the shorter duration of the intervention and the lower dosage of omega-3 supplied.

## Adverse Effects of Omega-3 Polyunsaturated Fatty Acids

The safety and tolerability of n-3 PUFAs have been the subject of ongoing controversy due to its potential as a nutritional therapeutic agent [121]. According to the United States Food and Drug Administration (FDA), marine n-3 PUFAs doses of up to 3 g/day are considered safe [122]. The most common adverse effects of omega-3 supplementation include nausea, gastrointestinal discomfort, and a fishy smell in the breath [123]. Furthermore, n-3 PUFAs have the ability to affect haemostasis, which may increase the risk of bleeding. They have an impact on thrombotic processes, such as the activation and aggregation of platelets [124]. When there are increased quantities of n-3 PUFAs, they compete with AA for cyclooxygenase enzymes. This conflict results in a decrease in the production of thromboxane A2, which is a strong promoter of platelet aggregation. At the same time, there is an increase in the production of thromboxane A3 from EPA, which is a less effective factor in promoting platelet aggregation. n-3 LC-PUFAs are also believed to influence the levels of some blood coagulation factors, however the findings from various studies are inconclusive [125].

## The Novel Perspectives of Omega-3 Polyunsaturated Fatty Acids Use

In accordance with the AHA, there is insufficient evidence to recommend the use of n-3 PUFAs supplementation in the general population without a high risk of CVD. Additionally, there is no consensus on the treatment approach for individuals at high risk of CVD.

Currently, there is not sufficient evidence to support the use of n-3 PUFAs supplementation for primary prevention of CVD in general [126].

The use of n-3 PUFAs can have positive effects on individuals with insulin resistance and glucose intolerance through many pathways. This is because the ability of n-3 PUFAs to lower triglyceride levels is linked to improvements in glucose metabolism [76]. Although numerous studies have shown diverse advantages of n-3 PUFA supplementation, its lasting impact on the prevention or treatment of diabetes mellitus is still a subject of debate [127]. Individuals with diabetes are advised to consume more foods that are rich in long-chain n-3 PUFAs EPA and DHA, such as fatty fish. This is because these acids have been found to have positive effects on lipoproteins, help prevent heart disease, and are associated with favorable health outcomes in observational studies. This recommendation aligns with the general public's advice [128, 129]. The available evidence does not definitively support the recommendation of n-3 PUFAs (EPA and DHA) supplementation for all individuals with diabetes as a means of preventing or treating cardiovascular events [4].

## Conclusions

Upon considering the antioxidant, anti-inflammatory, antilipidemic, and antiobesonic mechanisms of omega-3 fatty acid supplements, along with the findings from randomized controlled studies, it becomes evident that these supplements have beneficial effects in preventing and treating diabetes, as well as preventing and treating complications associated with diabetes, particularly cardiovascular diseases. Nevertheless, existing evidence does not substantiate the use of omega-3 (EPA and DHA) supplementation for patients with diabetes as a means of preventing or treating cardiovascular events. Individuals with diabetes are advised to consume fatty fish and foods rich in omega-3 fatty acids twice a week, as recommended for the general population, because of the positive impact on lipoproteins, prevention of CVD, and association with favorable health outcomes.

## Data Availability

No datasets were generated or analysed during the current study.
